# Study on the Effects of the Composite Addition of Al–5Ti–0.8C and La on the Microstructure and High-Temperature Mechanical Properties of ZL205A Alloy

**DOI:** 10.3390/ma15207087

**Published:** 2022-10-12

**Authors:** Yu Li, Guowei Zhang, Jingwei Niu, Hong Xu

**Affiliations:** School of Materials Science and Engineering, North University of China, Taiyuan 030051, China

**Keywords:** composite addition of Al–5Ti–0.8C and La, ZL205A alloy, microstructure, high-temperature strengthening

## Abstract

The effects of Al–5Ti–0.8C and the rare-earth element La on the microstructure and high-temperature mechanical properties of ZL205A alloy were investigated. We found that the grains of 0.1%La + 0.3%Al–5Ti–0.8C alloy were fine, the morphology of the as-cast Al_2_Cu phase was fragmented, and the precipitated phase was fine after T5 treatment. In particular, the high-temperature mechanical properties of 0.1%La + 0.3%Al–5Ti–0.8C alloy were significantly improved above 250 °C. The reason for the increase in high-temperature plasticity was attributed to the Al–La phase and the TiC particles, which refined the grains and reduced the tendency for intergranular fracture at high temperatures. More importantly, the high-temperature strengthening mechanism of the Al–5Ti–0.8C master alloy compounded with La was a result of the TiC introduced by the Al–5Ti–0.8C alloy, and the Al_11_La_3_ formed by the addition of La refined the grains in the matrix, promoted the precipitation of the needle-like θ’(Al_2_Cu) phase, reduced the size of the θ’(Al_2_Cu) phase, decreased the PFZ (Precipitation Free Zone), and increased the θ’(Al_2_Cu) phase number, hindering dislocation and grain boundary motion.

## 1. Introduction

Al–Si alloy is one of the main materials for manufacturing engine blocks and cylinder heads. With the increase in the power density of automobile engines, cylinder blocks and cylinder heads need to bear higher mechanical and thermal loads. However, the performance of common Al–Si casting alloys has reached its limit, and it is difficult to meet the requirements of the harsh working environment [1]. Cast aluminum–copper alloy is a high-strength aluminum alloy, and ZL205A aluminum alloy is one of the strongest cast aluminum–copper alloys, with good impact resistance. Under 250–300 °C working conditions, ZL205A alloy can also maintain a certain strength, and its high-temperature tolerance is improved compared to that of Al–Si alloy, making ZL205A alloy a preferred material for engine cylinder heads [2], but its long-term high-temperature working performance still cannot meet the use requirements. Therefore, it is necessary to carry out microstructure modification and property research to improve the mechanical properties of ZL205A alloy at high temperatures.

The performance of Al–Cu alloys is mainly improved through the addition of grain refiners and rare-earth elements. Fine grain size leads to the high strength and plasticity of the Al–Cu alloys. The most commonly used grain refiner for aluminum alloys is Al–Ti–B in industrial production, but TiB_2_ particles are large and aggregated easily. An Al–Ti–C grain refiner overcomes the above disadvantages of Al–Ti–B, since TiC particles are smaller than TiB_2_ particles, have a lower tendency to aggregate, and are not poisoned by elements such as Zr, Cr, Mn, etc. [3,4]. Therefore, Al–Ti–C is considered to be a promising grain refining agent [5,6]. Wang et al. [7] found that 0.2 wt.% Al–5Ti–0.75C has a good effect on the refinement of Al–5Cu alloy; the average grain size decreased from about 1000 to 50 μm. Kumar and Bichler [8] studied the effects of TiC on the microstructure and mechanical properties of the B319 alloy. The results showed that TiC refines the microstructure of the alloy and promotes the separation of the eutectic. With the increase in TiC content, the secondary dendrite spacing of the alloy decreases, and the hardness and yield strength of the as-cast alloy increase. Yang et al. [9,10] and Tian et al. [11] from Jilin University studied the effects of TiC particles on the high-temperature properties of aluminum–copper alloys and analyzed the reasons. The results showed that TiC can refine the aluminum alloy grains and that, at high temperatures, due to the large amount of TiC dispersed in the matrix, the thermal properties are relatively stable, which can hinder the dislocation movement and improve the high-temperature performance of the material.

Alloying with rare-earth elements is a common method to improve the high-temperature mechanical properties of aluminum–copper alloys. This method not only eliminates trace impurity elements in the melt [12], but also improves the heat resistance of the alloy via fine grain strengthening and precipitation strengthening [13,14,15]. Yao et al. [16] studied the effects of La on the high-temperature properties of cast aluminum–copper alloys, and the results showed that La could significantly improve the tensile strength and creep resistance of the alloys at high temperatures. Zhang et al. [17] and Jiang et al. [18] studied the effects of La on the interaction between the tensile properties and electrical properties of aluminum alloy. The results showed that the addition of La can promote the refinement of α-Al grains and improve the morphology of the Al_13_Fe_4_ intermetallic phase. La also plays a role in purifying aluminum alloys. Mahmoud et al. [19] studied the effects of the addition of 5.0 wt.% La on the low cooling and rapid solidification process and tissue morphology of Al–Si–Cu alloys. The results showed that the rare-earth element La was able to modify the eutectic morphology of the Al–Si alloy by forming compounds with Si at a certain temperature, improving the material properties. Zhang et al. [20] added different contents of La to an Al–6Si alloy and carried out solidification experiments. The results showed that the grain refinement of α-Al and the modification mechanism of eutectic Si by La were due to the increase in the heterogeneous nucleation rate of α-Al and the increase in the number of La atoms in eutectic Si, respectively.

It has been shown that the addition of Al–Ti–C and La can improve the fluidity of ZL205A alloy [21] and refine the grains of pure aluminum [22]. However, can the composite addition of Al–Ti–C master alloy and the rare-earth element La improve the high-temperature performance of ZL205A? If it can be improved, which is better for the high temperature performance of ZL205A: the effect of composite addition of Al–Ti–C master alloy and La, or single addition? Answers to the above issues have not been reported to date.

In this paper, the effects of the Al–5Ti–0.8C and La composite on the microstructure and high-temperature mechanical properties of ZL205A alloy were investigated, with the underlying mechanisms clarified.

## 2. Materials and Methods

Pure Al, pure Cd, Al–50%Cu, Al–4%V, Al–10%Mn, Al–4%Zr, Al–4%Ti–1%B, Al–10%La, and Al–5%Ti–0.8%C master alloys (Shenyang aerospace metal, Shenyang, China) were used to prepare the ZL205A alloy. The chemical composition of the ZL205A alloy was 4.95% Cu, 0.59% Mn, 0.17% Ti, 0.11% Cd, 0.12% V, 0.14% Zr, and 0.06% B balanced with Al (all compositions are in wt.%). To investigate the effects of the composite addition of Al–5Ti–0.8C and La, alloys with single addition of 0.3%Al–5Ti–0.8C, single addition of 0.1%La, and an unmodified alloy were also prepared. Pure Al and master alloys were melted in a resistance furnace (Shanghai Shiyan Electric Furnace, Shanghai, China), and Cd, Al-10La and Al–5Ti–0.8C were added at 780 °C; the temperature was lowered to 730 °C for degassing with C_2_Cl_6_, and then the melt was poured into a Y-shaped metal mold at a temperature of 710 °C. The sampling size was 155 × 35 × 25 mm^3^.

Four alloys were cast five times each. The specimens were subjected to T5 heat treatment. They were solution-treated at 538 ± 5 °C for 14 h, quenched in water at 50 °C, aged at 155 ± 5 °C for 8 h, and air-cooled. Grain size measurements were performed using the Micro-image Analysis and Process metallographic analysis software (ProImaging V1.0, 2021, Zeiss, Oberkohen Municipality, German). The microstructure was characterized using a SU-5000 (with an energy-dispersive spectrometer (EDS)) scanning electron microscope (SU5000, Tokyo, Japan) operated at 20 kV, with elemental distribution analyzed by EDS. The hardness of the alloy was determined using a 210HBS-3000 digital Brinell hardness tester (Laizhou Huayin, Laizhou, China) with a 5 mm indenter diameter. Tensile tests were carried out at room temperature and high temperatures with a strain rate of 0.001/s using an INSTRON-3382 universal electronic tensile tester (Instron, Boston, MA, USA). The high-temperature tensile specimens were characterized using a Philips TECNAI 20 transmission electron microscope (PHILIPS, Amsterdam, Netherlands). High-temperature aging was carried out at 250 and 300 °C for different times, as shown in Table 1. The evolution of the microhardness was measured using a Vickers hardness tester (Laizhou Huayin, Laizhou, China).

## 3. Results and Discussion

### 3.1. Microstructure

#### 3.1.1. Grain Size Analysis

**Figure 1:**
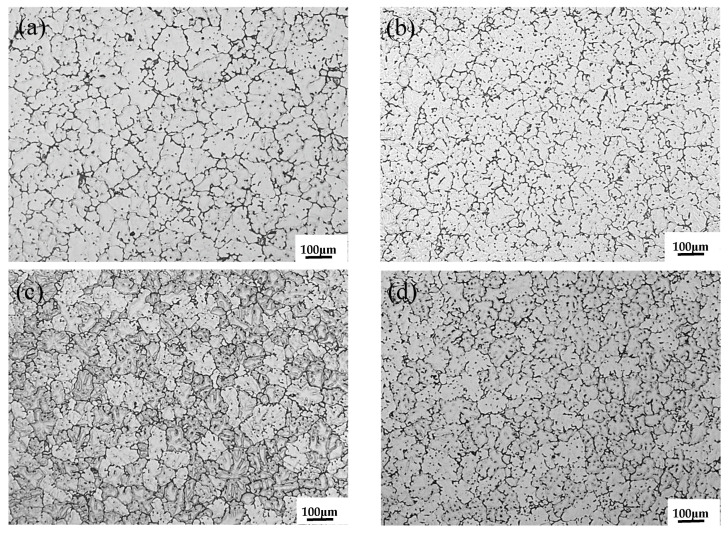
As-cast microstructure of (**a**) ZL205A alloy, (**b**) ZL205A + 0.1%La alloy, (**c**) ZL205A + 0.3%Al–5Ti–0.8C alloy, and (**d**) ZL205A + 0.1%La + 0.3%Al–5Ti–0.8C alloy.

**Figure 2:**
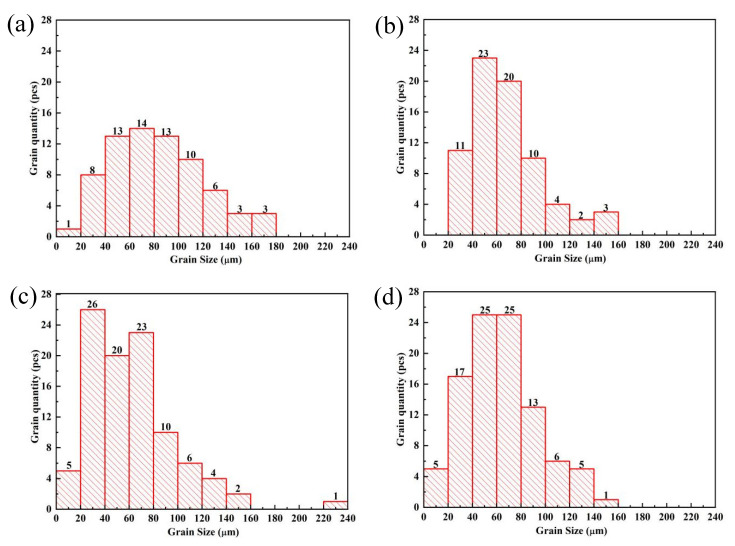
Histogram of the grain size distribution of (**a**) ZL205A alloy, (**b**) ZL205A + 0.1%La alloy, (**c**) ZL205A + 0.3%Al–5Ti–0.8C alloy, and (**d**) ZL205A + 0.1%La + 0.3%Al–5Ti–0.8C alloy.

The microstructure of the ZL205A alloy with composite addition of 0.1%La + 0.3%Al–5Ti–0.8C and single addition of 0.3%Al–5Ti–0.8C or 0.1%La is shown in Figure 1. The addition of Al–5Ti–0.8C and La to the ZL205A alloy resulted in significant refinement of the grains. The grain size was measured by the line-intercept method, and the results are shown in Figure 2.

The average grain sizes are shown in Table 2. The average grain size of the ZL205A alloy was 82 μm. After adding Al–5Ti–0.8C and La, the average grain size decreased significantly—to 62 μm for the alloy with 0.3%Al–5Ti–0.8C, 68 μm for the alloy with 0.1%La, and 61 μm for the alloy with 0.1%La + 0.3%Al–5Ti–0.8C. The refining effect was equivalent to that of the Al–5Ti–0.8C master alloy, but better than that of La.

#### 3.1.2. Microstructure Analysis

**Figure 3:**
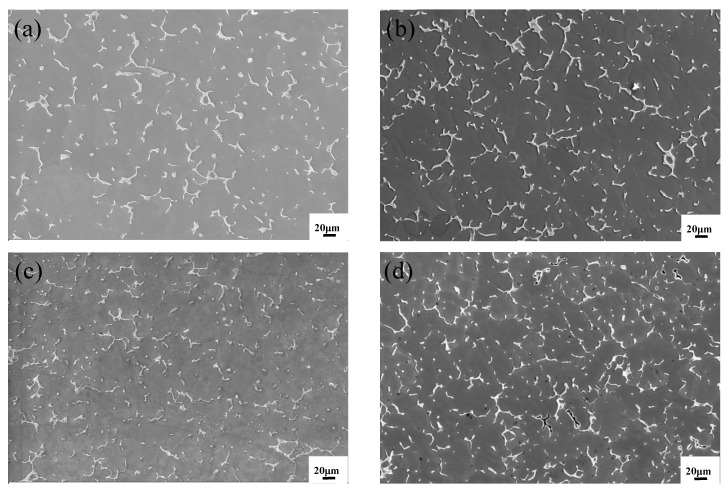
SEM (Scanning Electron Microscope) diagram of the cast state of (**a**) ZL205A alloy, (**b**) ZL205A + 0.1%La alloy, (**c**) ZL205A + 0.3%Al–5Ti–0.8C alloy, and (**d**) ZL205A + 0.1%La + 0.3%Al–5Ti–0.8C alloy.

The as-cast microstructures of ZL205A alloys with 0.1%La + 0.3%Al–5Ti–0.8C and single addition of 0.3%Al–5Ti–0.8C and 0.1%La are shown in Figure 3. It can be seen from Figure 3a that the matrix phase of the unmodified alloy is α-Al, the θ(Al_2_Cu) is discontinuously distributed on the grain boundary, and the structure is coarse, splitting the matrix. When 0.1%La is added alone, the θ phase structure is partially fragmented, as shown in Figure 3b. When 0.3%Al–5Ti–0.8C is added alone and 0.3%Al–5Ti–0.8C + 0.1%La are added in combination, the θ phase is obviously fragmented, from network to point- or worm-like, as shown in Figure 3c,d.

**Figure 4:**
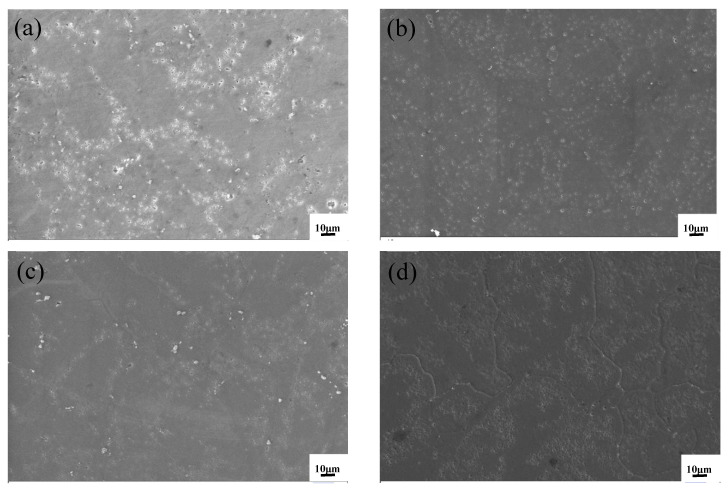
Microstructure of the alloys after aging: (**a**) ZL205A alloy, (**b**) ZL205A + 0.1%La alloy, (**c**) ZL205A + 0.3%Al–5Ti–0.8C alloy, and (**d**) ZL205A + 0.1%La + 0.3%Al–5Ti–0.8C alloy.

Figure 4 shows the microstructure of ZL205A alloy with composite addition of 0.1%La + 0.3%Al–5Ti–0.8C and single additions of 0.3%Al–5Ti–0.8C and 0.1%La after T5 heat treatment. For the ZL205A, the undissolved phase was also larger and distributed along the grain boundaries, and the precipitated phase was coarse. When 0.3%Al–5Ti–0.8C was added (Figure 4b), the precipitated phase was obviously refined and uniformly dispersed. For the ZL205A alloy with 0.1%La (Figure 4c), the precipitated phase was finer and more uniformly distributed than the unmodified alloy. When 0.1%La + 0.3%Al–5Ti–0.8C (Figure 4d) were added, the precipitated phase was smaller than that of the alloy with Al–5Ti–0.8C and La.

### 3.2. Room-Temperature Mechanical Properties

**Figure 5:**
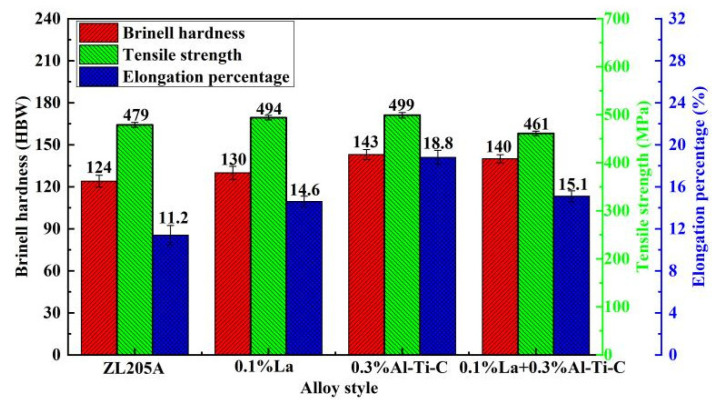
Mechanical properties of the alloys after T5 heat treatment.

The mechanical properties of the alloys are shown in Figure 5. After T5 heat treatment, the hardness of the alloy with the addition of 0.1%La + 0.3%Al–5Ti–0.8C was 140 HBW, and the tensile strength and elongation were 461 MPa and 15.1%, respectively. The hardness and elongation were 12.9% and 34.8% higher than those of the unmodified alloy, respectively, 7.7% and 3.4% higher than that of the alloy with 0.1% La, respectively, but the tensile strength was lower than that of the unmodified alloy and the alloys with 0.1%La or 0.3%Al–5Ti–0.8C.

**Figure 6:**
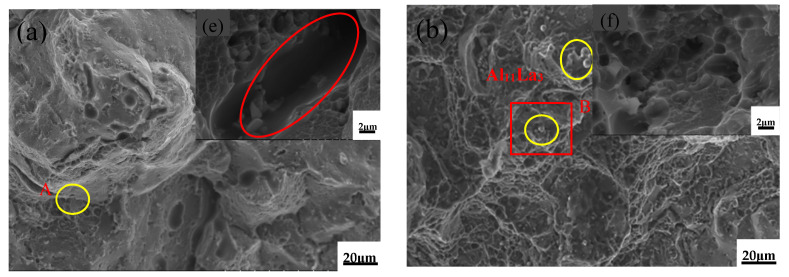

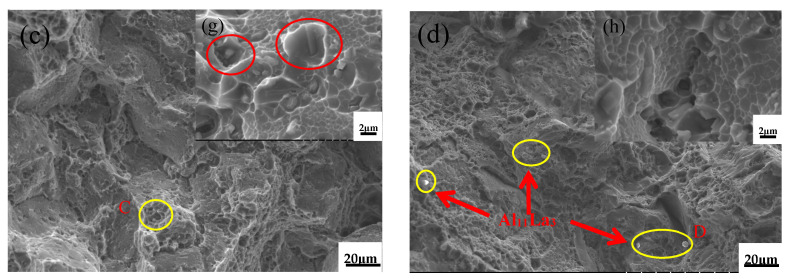
Fracture surface of the T5 heat-treated alloys after room-temperature tensile tests: (**a**) ZL205A alloy, (**b**) ZL205A + 0.1%La alloy, (**c**) ZL205A + 0.3%Al–5Ti–0.8C alloy, (**d**) ZL205A + 0.1%La + 0.3%Al–5Ti–0.8C alloy, (**e**) Enlarged image of area A, (**f**) Enlarged image of area B, (**g**) Enlarged image of area C, (**h**) Enlarged image of area D.

**Figure 7:**
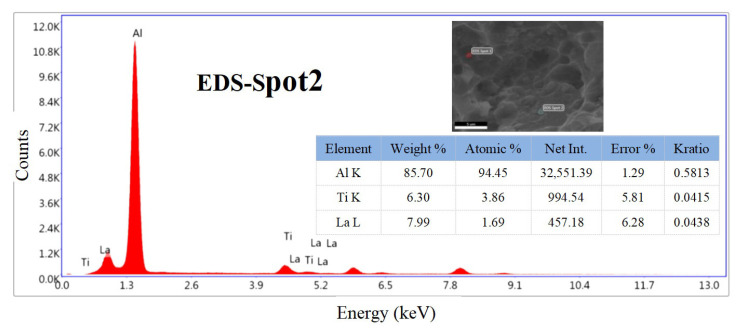
EDS results of the red area B in Figure 6b.

Figure 6 shows the fracture surface of the T5 heat-treated alloys after room-temperature tensile tests. The unmodified alloy has a large number of fractures along the grain and a small number of shallow and small dimples near the grain boundaries, as shown in Figure 6a. After the addition of 0.1%La, the fracture surface of the alloy changed significantly, with an increase in the number of small and deep dimples, and a small amount of intergranular fracture, as shown in Figure 6b, while a small amount of fine Al–La phase was observed on the fracture surface (the EDS result of the red area B in Figure 6b is shown in Figure 7). When 0.3%Al–5Ti–0.8C was added, the number of dimples increased and the size decreased significantly. When adding 0.1%La + 0.3%Al–5Ti–0.8C, more and smaller dimples were observed, and a small amount of Al–La phase appeared in a round cake shape.

### 3.3. High-Temperature Mechanical Properties

**Figure 8:**
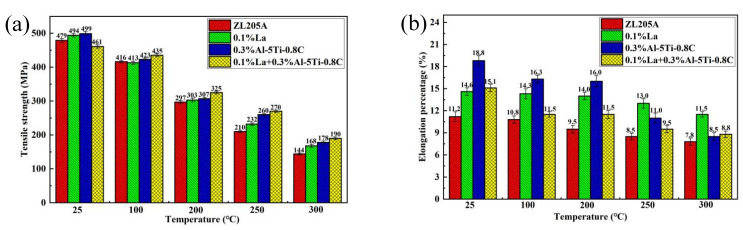
High-temperature mechanical properties of ZL205A alloy after T5 heat treatment: (**a**) tensile strength; (**b**) elongation.

The mechanical properties of the ZL205A alloy with the addition of La and Al–5Ti–0.8C at different temperatures are shown in Figure 8. The tensile strength of the ZL205A alloy at 300 °C was 144 MPa and the elongation was 7.8%; the tensile strength of the alloy with the addition of 0.1%La increased by 16.7% and the elongation by 47.4% compared to the unmodified alloy; the addition of 0.3%Al–5Ti–0.8C increased the tensile strength at 300 °C by 23.6% and the elongation by 8.97% compared with the unmodified alloy; the tensile strength at 300 °C increased by 31.9% and the elongation increased by 12.8% compared with the unmodified alloy when adding 0.1%La + 0.3%Al–5Ti–0.8C, and in this treatment the tensile strength increased by 13.1% and elongation decreased by 23.5% compared to the alloy with 0.1%La, while the tensile strength increased by 6.7% and elongation increased by 3.5% compared to the alloy with 0.3%Al–5Ti–0.8C. It can be seen that the composite of Al–5Ti–0.8C and the rare-earth element La improves the high-temperature strength of ZL205A significantly.

In Figure 8b, the elongation of the alloy decreases with increasing temperature. This is because V, Ti, Zr, and other elements in the ZL205A alloy form insoluble Al_3_V, Al_3_Ti, Al_3_Zr, and other intermetallic compounds, which are easily enriched on the grain boundaries. As the temperature increases, the grains coarsen and the grain boundaries begin to soften, weakening the bonding between the grain boundaries. Intermetallic compounds enriched at grain boundaries hinder grain boundary deformation, causing cracks to propagate along fragile grain boundaries. With the increase in temperature, the fracture dimples become larger and the elongation decreases.

**Figure 9:**
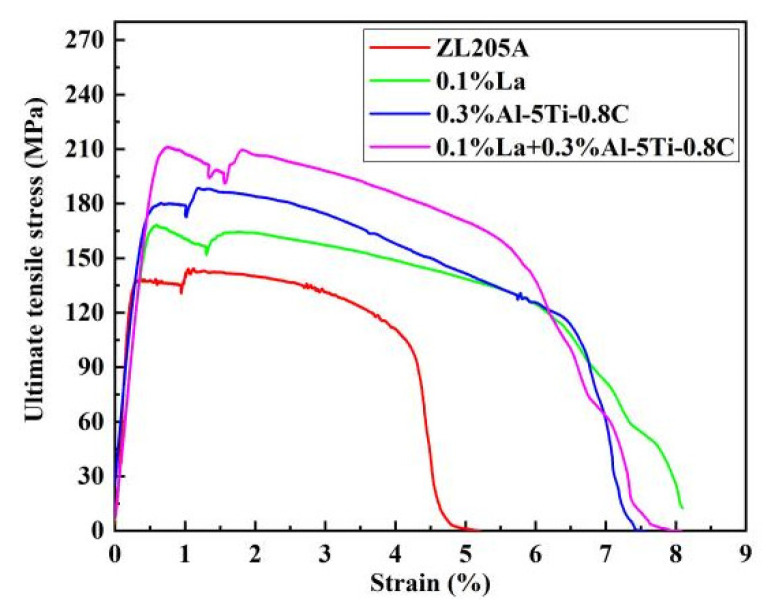
Tensile stress–strain curve of the alloys at 300 °C.

Figure 9 shows the tensile stress–strain curves of the ZL205A alloy with the additions of La and Al–5Ti–0.8C at 300 °C. Work hardening occurred during deformation, along with softening caused by dynamic recrystallization, and the ZL205A alloy with the addition of 0.1%La + 0.3%Al–5Ti–0.8C had the most significant increase in high-temperature tensile strength.

**Figure 10:**
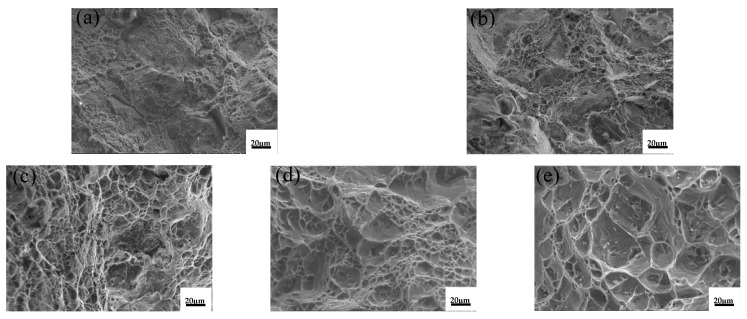
Tensile fracture morphology of ZL205A alloy with 0.1%La + 0.3%Al–5Ti–0.8C at different temperatures: (**a**) 25 °C, (**b**) 100 °C, (**c**) 200 °C, (**d**) 250 °C, and (**e**) 300 °C.

The tensile fracture morphology of the ZL205A alloy with 0.1%La + 0.3%Al–5Ti–0.8C added at different temperatures is shown in Figure 10. At room temperature (25 °C), the dimples at the section were small and shallow, there was no obvious porosity, and the plasticity of the material was good. When the temperature increased, the dimples at the section gradually became larger. When the temperature exceeded 250 °C, the dimples started to appear along with clearly torn ridges, which may represent the boundaries of grains and subgrains. When the temperature reached 300 °C, the fracture showed an obvious tearing ridge, and the dimples were large and shallow, indicating poor plasticity of the material. In short, as the temperature increased, the grain boundary bonding of the ZL205A alloy weakened due to grain boundary softening, and when the material was broken, it was broken along the fragile grain boundary, and there were obvious tearing ridges and partial intergranular fracture characteristics, which reduced the plasticity of the material.

**Figure 11:**
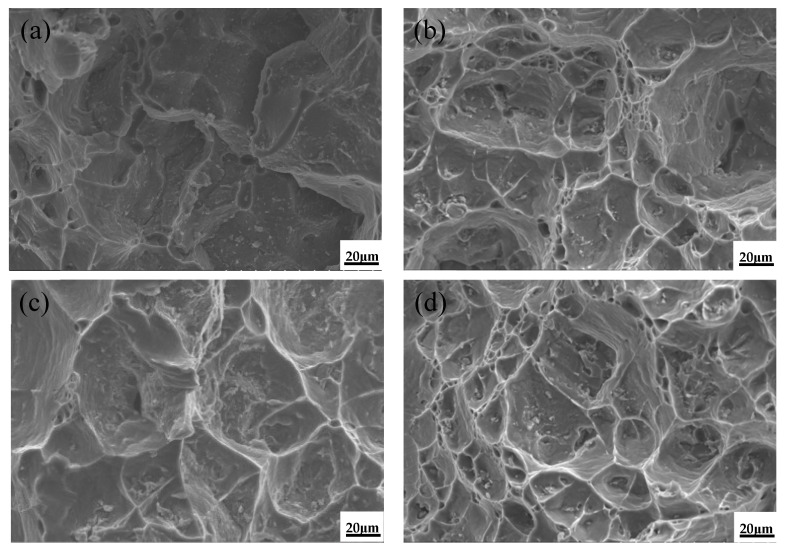
Tensile fracture morphology of T5 heat-treated alloy at 300 °C: (**a**) ZL205A alloy, (**b**) ZL205A + 0.1%La alloy, (**c**) ZL205A + 0.3%Al–5Ti–0.8C alloy, and (**d**) ZL205A + 0.1%La + 0.3%Al–5Ti–0.8C alloy.

The fracture surface of the unmodified alloy and the alloy with the addition of La and Al–5Ti–0.8C at 300 °C is shown in Figure 11. The fracture of the unmodified alloy shows obvious tear ridges with large and shallow dimples. When 0.1%La was added, a large number of dimples with flat shapes and uniform distribution formed. When 0.3%Al–5Ti–0.8C was added, some dimples of larger size appeared, and the fracture along the grain boundaries was more obvious. The overall number of dimples in the alloy after adding 0.1%La was greater than that of the alloy with 0.3%Al–5Ti–0.8C, and the grain boundaries’ fracture was impeded, indicating that the plasticity of the ZL205A alloy is more obviously enhanced by La. After adding 0.1%La + 0.3%Al–5Ti–0.8C, the dimples were deeper and more evenly distributed, and the surface was flat, with some particles in the dimples.

### 3.4. High-Temperature Strengthening Mechanism

**Figure 12:**
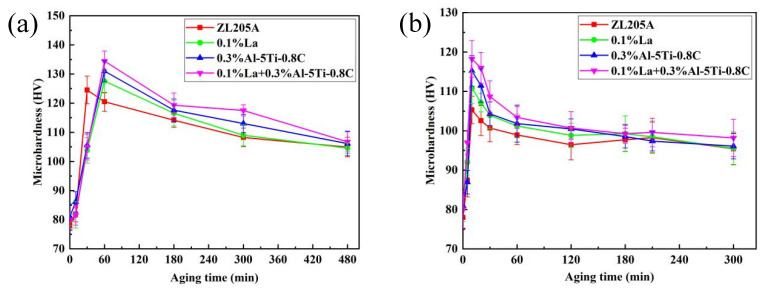
Isothermal age–hardening curves of ZL205A alloy at (**a**) 250 °C and (**b**) 300 °C.

To explain the high-temperature strengthening of ZL205A alloy with the addition of Al–5Ti–0.8C and La composite, the age–hardening curves of the alloy at 250 and 300 °C were measured, as shown in Figure 12. At 250 °C, the time to reach the aging peak changed between the unmodified alloy and the alloy with the addition of 0.1% La and 0.3%Al–5Ti–0.8C. The time to reach peak hardness for the unmodified alloy was 30 min, with a peak hardness of 125HV. The aging time to peak hardness for the alloys with 0.1%La and 0.3%Al–5Ti–0.8C was 1 h, which was 30 min longer than that for the unmodified alloy. The peak hardness for the alloy with 0.1%La was 128HV, while for the alloy with 0.3%Al–5Ti–0.8C the peak hardness was 131HV, and the peak hardness of the alloy with 0.1%La + 0.3%Al–5Ti–0.8C was 135HV.

The age–hardening curves at an aging temperature of 300 °C are shown in Figure 12b. The time to peak hardness at 300 °C was 10 min for both the unmodified alloy and the alloy with 0.1%La and 0.3%Al–5Ti–0.8C. The peak hardness was 105HV for the unmodified alloy, 111HV for the alloy with 0.1%La, 115HV for the alloy with 0.3%Al–5Ti–0.8C, and 118HV for the composite. The peak hardness of the alloy with 0.3%Al–5Ti–0.8C and 0.1%La was 118HV. The peak hardness of the alloy with 0.1%La + 0.3%Al–5Ti–0.8C was higher than that of the single-added alloys at aging temperatures of 250 °C and 300 °C. This could also explain the significant improvement in the high-temperature mechanical properties of the alloy with 0.1%La + 0.3%Al–5Ti–0.8C at 250 and 300 °C.

Revealing the mechanism of high-temperature strengthening of the Al–5Ti–0.8C intermediate alloy with rare-earth La composite requires an in-depth analysis of the aging precipitation phase of ZL205A. Excess vacancies in the ZL205A alloy play an important role in the formation of θ’(Al_2_Cu) precipitates [23,24,25], and the density of vacancies determines the amount of θ’(Al_2_Cu) precipitates. On the one hand, at the solution treatment temperature, vacancies present in the matrix migrate to the grain boundaries during quenching, indicating that grain size affects the precipitation kinetics and that the number of grain boundaries increases significantly, providing more sites for precipitate nucleation. On the other hand, θ’(Al_2_Cu) precipitates mainly as precipitation, and the diffusion of atoms is an important factor affecting precipitation. The grain boundary is a surface defect, and the defects near the grain boundary are associated with higher energy, while dislocation lines are also plugged near the grain boundary. The θ’(Al_2_Cu) is easily nucleated at the grain boundary due to the short distance and low energy required for atomic diffusion near the grain boundary.

**Figure 13:**
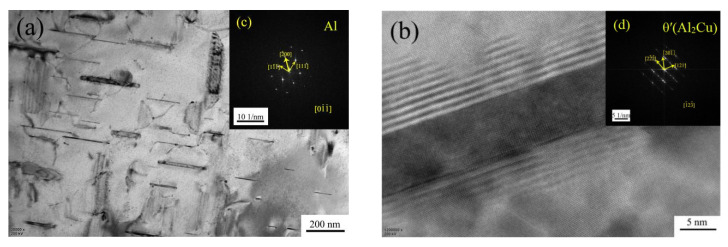
Bright-field image and HRTEM image of the unmodified ZL205A alloy after T5 treatment: (**a**) bright-field image, (**b**) θ’-phase HRTEM image, (**c**) diffraction pattern of the α phase, and (**d**) diffraction pattern of the θ’ phase.

Figure 13 shows the TEM (Transmission Electron Microscope) bright-field image and HRTEM (High Resolution Transmission Electron Microscope) image of the θ’ phase of the unmodified ZL205A alloy after T5 heat treatment, and the main phases in the matrix are α(Al) and θ’(Al_2_Cu), as marked by the diffraction pattern. Figure 13b shows the morphology of θ’(Al_2_Cu) under HRTEM, with a needle-like shape.

**Figure 14:**
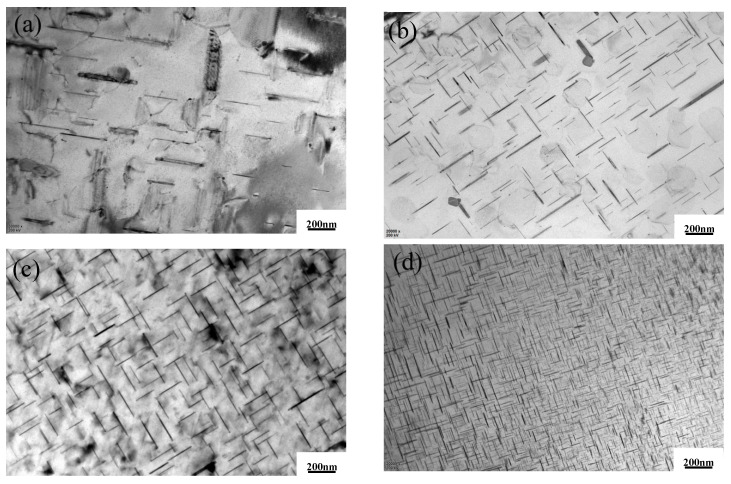
TEM bright-field images of (**a**) ZL205A alloy, (**b**) ZL205A + 0.1%La alloy, (**c**) ZL205A + 0.3%Al–5Ti–0.8C alloy, and (**d**) ZL205A + 0.1%La + 0.3%Al–5Ti–0.8C alloy after T5 treatment.

Figure 14 shows the TEM bright-field image of the ZL205A alloy with Al–5Ti–0.8C compounded with La. It can be seen from Figure 14b that the amount of the θ’(Al_2_Cu) phase increases and the precipitated phase becomes smaller for the ZL205A alloy with the addition of La, indicating that the addition of La inhibits the growth of the θ’(Al_2_Cu) phase but the refinement effect is not as good as that of the alloy with the addition of Al–5Ti–0.8C. Figure 14d shows the ZL205A alloy with 0.1%La + 0.3%Al–5Ti–0.8C, where the amount of θ’(Al_2_Cu) in the matrix is significantly increased and homogeneously distributed in the matrix, and the precipitates are fine, which is consistent with the results of the high-temperature tensile tests. The increase in strength of the ZL205A alloy at high temperatures is partially due to fine grain strengthening, and can be partially attributed to the refinement and increase in the amount of the θ’(Al_2_Cu) phase.

**Figure 15:**
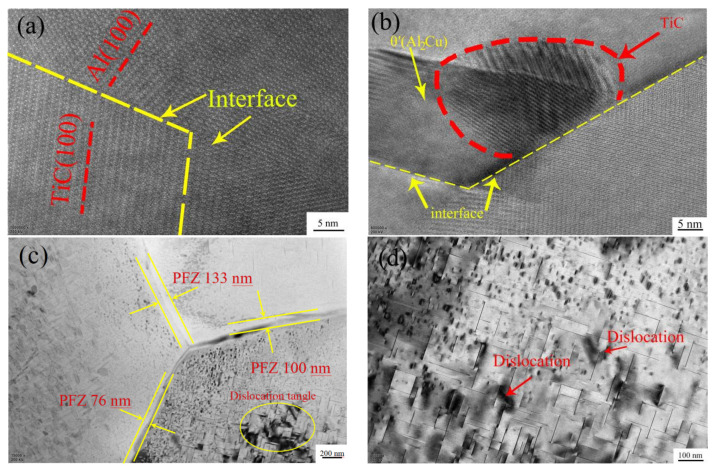
TEM bright-field image and HRTEM image of ZL205A alloy with 0.3%Al–5Ti–0.8C + 0.1%La: (**a**) the interface of Al and TiC, (**b**) θ’(Al_2_Cu)-dependent TiC precipitation, (**c**) PFZ (Precipitation Free Zone) area, and (**d**) dislocations near θ’(Al_2_Cu).

Figure 15 shows the TEM bright-field image and HRTEM image of the ZL205A alloy with 0.3%Al–5Ti–0.8C + 0.1%La. It can be seen that the TiC in the ZL205A alloy with 0.1%La + 0.3%Al–5Ti–0.8C has a good matching relationship with the Al matrix, as shown in Figure 15a, and there is also some θ’ near the TiC particles’ θ’(Al_2_Cu), as shown in Figure 15b. The PFZ (Precipitation Free Zone) becomes significantly narrower in the region where θ’(Al_2_Cu) precipitates intensively, as shown in Figure 15c, which indicates that the addition of Al–5Ti–0.8C to the alloy refines the grains and increases the number of grain boundaries under the action of [Ti] and TiC, increasing the number of nucleation sites at the grain boundaries during aging and promoting the precipitation of θ’(Al_2_Cu), with TiC as the matrix and the precipitate relying on TiC particles. The above factors accelerate the precipitation of the θ’(Al_2_Cu) phase in the matrix and refine the θ’(Al_2_Cu) phase.

**Figure 16:**
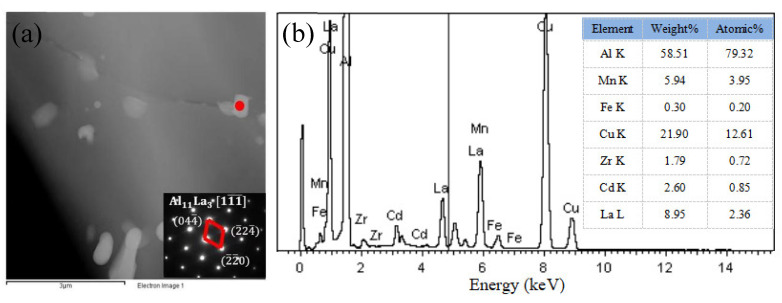
Al–La phase in ZL205A + 0.3%Al–5Ti–0.8C + 0.1%La alloy (T5 heat treatment): (**a**) the electron diffraction spots (dark field), (**b**) the electron energy spectrum.

Due to the addition of the rare-earth element La, a certain amount of Al–La phase is formed in the ZL205A alloy after aging. In order to confirm the microstructure of the Al–La phase, transmission electron microscopy and electron energy spectrum analysis were carried out. Figure 16 shows the electron energy spectrum results and electron diffraction spots (dark field) of the Al–La phase. Through analysis, it was confirmed that the block lanthanum-rich phase is an Al_11_La_3_ phase and its size is submicron. This phase is dispersedly distributed in the matrix and acts as a pinning dislocation.

Based on the Hall–Petch relationship [26], the relationship between the grain size and the increase in strength *Δσ*_HP_ can be described by Equation (1), where *k* is the Hall–Petch coefficient and *d* is the average grain size:

(1) $$\Delta \sigma_{\mathrm{HP}}=k(d_{\mathrm{Alloy}}^{-1/2}-d_{\mathrm{ZL}205A}^{-1/2})$$

In addition, the composite addition of 0.1%La + 0.3%Al–5Ti–0.8C to the ZL205A alloy increases the amount of θ’(Al_2_Cu) phase precipitation, which is also key to improving the high-temperature performance. The θ’(Al_2_Cu) phase is uniformly distributed in the matrix (Figure 14d), and the uniform and fine θ’(Al_2_Cu) phase plays an important role in hindering dislocation motion. Therefore, according to the Orowan mechanism of dislocation motion [27], the reinforcing effect of precipitates can be expressed by Equations (2)–(4):

(2) $$\Delta \sigma_{OR}=M\frac{0.4Gb}{\pi \lambda }\frac{\ln(\frac{2r}{b})}{\sqrt{1-\nu }}$$

(3) $$\lambda =\left({\left(\frac{\pi }{f_{p}}\right)}^{\frac{1}{2}}-2\right)r$$

(4) $$r=\sqrt{\frac{1}{6}}d_{p}$$

where *Δσ_OR_* is the contribution of strengthening from precipitates, *f*_p_ is the volume fraction of the θ’(Al_2_Cu) phase, *d*_p_ is the average radius of the precipitates, *r* is the average thickness of the precipitates, *G* is the shear modulus, *b* is the Burgers vector, *v* is the Poisson’s ratio, *M* is the Taylor factor, and *G*, *b*, *v*, and *M* are constants. According to the equations, it can be seen that the strength increment *Δσ_OR_* increases with the decrease in the average radius of the precipitates *d*_p_ and the increase in the volume fraction *f*_p_ of the θ’(Al_2_Cu) phase [28]. As shown in Figure 14, the increase in the number of θ’(Al_2_Cu) precipitates and the decrease in size are essential to improve the room-temperature and high-temperature properties of the ZL205A alloy with 0.3%Al–5Ti–0.8C + 0.1%La.

In summary, through the composite strengthening of Al–5Ti–0.8C and La, the TiC introduced by the Al–5Ti–0.8C alloy and the Al_11_La_3_ formed by the addition of La refine the grains in the matrix, and the resulting grain boundary enrichment dislocation and vacancy concentration increase provide conditions for the nucleation of the θ’(Al_2_Cu) phase, accelerate the precipitation of the θ’(Al_2_Cu) phase, refine the θ’(Al_2_Cu) phase, and narrow the PFZ region. During high-temperature deformation, dislocations recover, grain boundaries move, and grains recrystallize. The large amounts of fine θ’(Al_2_Cu) phases, TiC, and Al_11_La_3_ in the matrix pin dislocations and hinder the movement of grain boundaries. In addition, TiC and Al_11_La_3_ can provide a substrate for recrystallized grains and inhibit the growth of recrystallized grains. Under the combined action of the above factors, the high-temperature performance of the ZL205A alloy is improved.

## 4. Conclusions

1. The grains of the 0.1%La + 0.3%Al–5Ti–0.8C alloy are fine, the morphology of the as-cast Al_2_Cu phase is fragmented, and the precipitated phase is fine after T5 treatment. The high-temperature strength of the 0.1%La + 0.3%Al–5Ti–0.8C alloy increases significantly without decreasing the elongation above 250 °C. After T5 heat treatment, the tensile strength and elongation at 300 °C can be as high as 190 MPa and 8.8%, respectively. Compared with unmodified alloy and single additions, the maximum increase in tensile strength is 31.9%, and the elongation is slightly increased.
2. Compound addition of 0.1%La + 0.3%Al–5Ti–0.8C increases the high-temperature plasticity of ZL205A due to the Al–La phase and the TiC particles, which refine the grains and reduce the tendency for intergranular fracture at high temperatures.
3. The TiC introduced by the Al–5Ti–0.8C alloy and the Al_11_La_3_ formed by the addition of La refine the grains in the matrix, promote the precipitation of the needle-like θ’(Al_2_Cu) phase, reduce the size of the θ’(Al_2_Cu) phase, decrease the PFZ, and increase the θ’(Al_2_Cu) phase number, hindering dislocation and grain boundary motion.

## Figures and Tables

**Table 1:** High-temperature aging process of ZL205A alloy.

Aging Temperature (°C)	Aging Time (min)									
250	-	10	-	30	60	-	180	-	300	480
300	5	10	20	30	60	120	180	210	300	-

**Table 2:** Grain size of ZL205A alloy with the addition of Al–5Ti–0.8C and La.

Specimen	Unmodified Alloy	0.3%Al–5Ti–0.8C Alloy	0.1%La Alloy	0.1%La + 0.3%Al–5Ti–0.8C Alloy
Average grain size/μm	82	62	68	61

## Data Availability

Not to public.
